# Lateral Carrier Diffusion in Ion-Implanted Ultra-Small Blue III-Nitride MicroLEDs

**DOI:** 10.1021/acsami.4c14784

**Published:** 2025-01-15

**Authors:** Julia Slawinska, Grzegorz Muziol, Anna Kafar, Czeslaw Skierbiszewski

**Affiliations:** Institute of High Pressure Physics, Polish Academy of Sciences, Warsaw 01-142, Poland

**Keywords:** microLED, III-nitride, carrier dynamics, diffusion, AR/VR

## Abstract

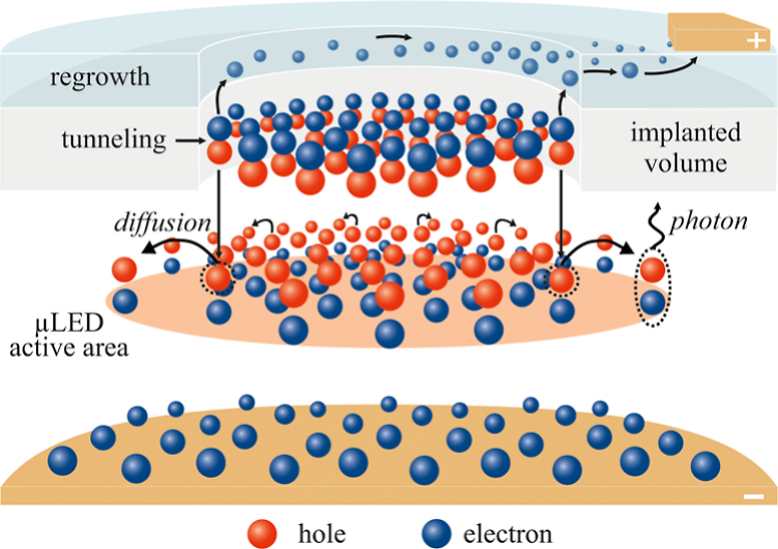

Ultrasmall micro-light-emitting diodes (μLEDs), sized below 10 μm, are indispensable to create the next-generation augmented and virtual reality (AR/VR) devices. Their high brightness and low power consumption could not only enhance the user experience by providing vivid and lifelike visuals but also extend device longevity. However, a notable challenge emerges: a decrease in efficiency with a reduced size. This study casts light on this critical issue by investigating the lateral carrier diffusion in ion-implanted μLEDs. The implanted area restricts the carrier injection and defines the μLED size to diameters of 10, 5, and 2 μm without introduction of nonradiative recombination centers in the quantum well area. We observed a drop of efficiency for smaller devices, similar to the case of conventional μLEDs with etched sidewalls. Electroluminescence of μLEDs was studied using a Gaussian beam telescope to analyze light intensity profiles and hence the spatial carrier distribution within the active region of μLEDs. Lateral diffusion length was determined to be 11.2 μm at *j* = 1 A/cm^2^ and decreased down to 2.4 μm for *j* = 1000 A/cm^2^. We explain the underlying mechanism behind the size-dependent efficiency observed in μLEDs, attributed to lateral carrier diffusion.

## 1 Introduction

Micro-light-emitting diodes (μLEDs) are promising candidates for the next generation display technology due to their attributes of high luminance, low energy demand, and long lifetime.^1^ Ultrasmall μLEDs, with lateral dimensions below 10 μm, find applications in technologies like smartwatches or augmented and virtual reality, where the demand for high pixel density is required.^2,3^ Unfortunately, reduced μLED size leads to a decline in the internal quantum efficiency (IQE) due to sidewall defects formed during fabrication. These defects act as nonradiative Shockley–Read–Hall (SRH) recombination centers, especially affecting ultrasmall μLEDs with their high surface-to-volume ratio.^4−9^ To address this issue, researchers have employed various techniques like sidewall passivation through atomic layer deposition,^10−12^ selective area growth,^13^ and current management using tunnel junctions (TJs).^14−17^ Ion implantation is also getting more popular and it was used both to passivate the sidewalls^18^ and to define the size of μLEDs.^19−21^ Applying a TJ on the top of the μLEDs facilitates conversion of electrical conductivity from n-type to p-type, leading to efficient hole injection into the LED structure.^22^ This approach allows us to substitute p-type semitransparent contacts, e.g., transparent conductive oxides with highly conductive n-type GaN, offering advantages in terms of light absorption and subsequent light extraction efficiency.^23^ Moreover, TJs not only enhance optical performance but also enable designing a monolithic stack of μLEDs each emitting a different color.^24−26^ Furthermore, one of the most critical parameters in μLEDs is lateral carrier diffusion length (*L*_d_). It plays a crucial role in characterizing the amount of carriers capable of accessing the sidewall region and undergoing nonradiative recombination, thereby decreasing IQE.^27^ It is commonly believed that in nitrides, *L*_d_ may be in the order of a few hundred nanometres, based on the short carrier lifetime (τ) measured during time-resolved photoluminescence (PL).^28−31^ However, recent work has shown that *L*_d_ can reach up to a few tens of microns in high-efficiency structures grown by metal–organic chemical vapor deposition on bulk GaN, as demonstrated by PL measurements.^32,33^

Until now, lateral diffusion has not been directly quantified in μLED structures. The reason is that diffusion cannot be assessed in standard μLEDs because carriers recombine nonradiatively on the sidewalls of the device. Moreover, it has not been investigated in LED structures used for μLED fabrication, but rather in test structures, such as single quantum wells (QWs) embedded in p–i–n diodes or undoped heterostructures, which are specially designed for PL measurements.^32,33^

**Figure 1:**
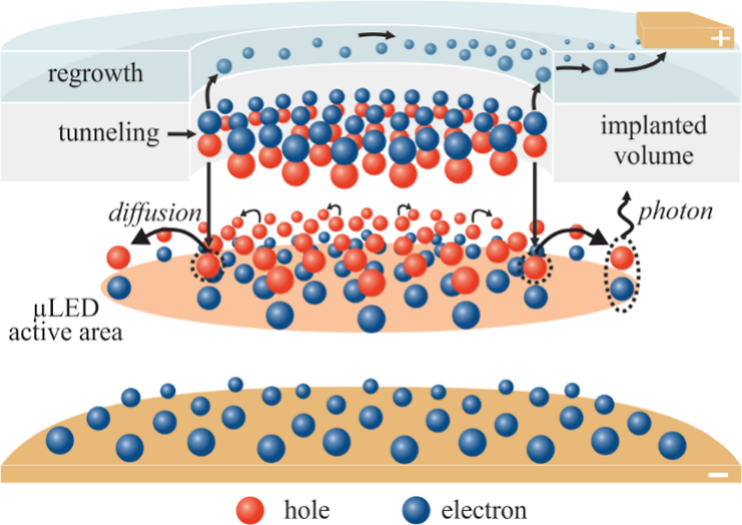
Scheme of carrier distribution inside ion-implanted TJ μLED. The unimplanted region defines the current injection area, thus determining the size of the μLED active area, which is also influenced by the lateral diffusion of carriers.

In this study, we investigate the influence of lateral carrier diffusion on the efficiency of electrically powered μLEDs. In comparison to PL, during electroluminescence (EL) measurements, the behavior of carriers can be influenced by the drift, created by the applied voltage. Ion-implanted μLEDs with TJs are well-suited for investigating lateral carrier transport during EL measurements due to their unique geometry. Here, the size is defined not by etching the sidewalls of the QWs but rather by modification of the electrical path. The scheme of carrier flow in ion-implanted μLED is shown in Figure 1. The electrical path is restricted to unimplanted regions. Due to changes in the conductivity caused by ion implantation, electrons tunnel only in the unimplanted region, resulting in holes being injected into the LED structure solely in these areas. Since the QW size is not limited by sidewalls (see Figure 1), the carriers injected into the QW can diffuse laterally over distances longer than the size of the designed device (the size of the unimplanted region). This allowed for the study of lateral carrier diffusion in the QWs.

## 2 Methods

**Figure 2:**
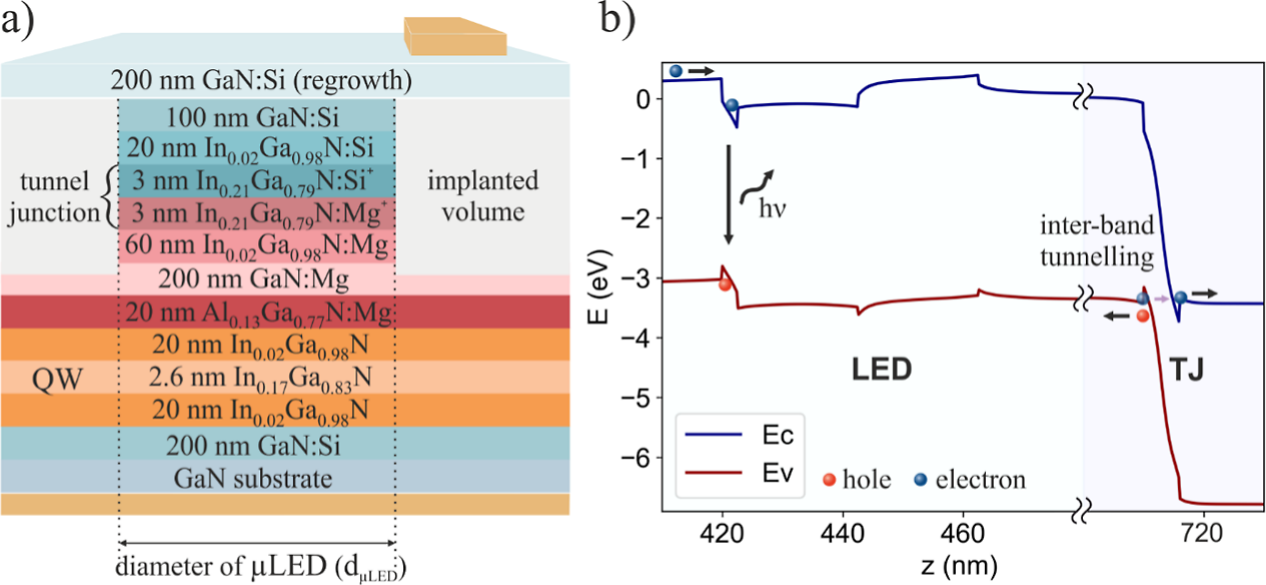
(a) Epitaxial structure of ion implanted TJ μLED with diameters 2, 5, and 10 μm (b) and energy band diagram under operating condition *V* = 3.2 V with the LED region, where radiative recombination occurs, and TJ region, where interband tunnelling is present.

We present ion-implanted μLEDs with a TJ grown by plasma-assisted molecular beam epitaxy (PAMBE) on bulk GaN substrates. The epitaxial structure is illustrated in Figure 2a. In order to study the influence of the size on the properties of μLEDs, we fabricated devices with diameters of 2, 5, and 10 μm, as well as 300 × 300 μm^2^ standard LEDs from the same wafer. The fabrication consists of five steps. (i) PAMBE growth of the TJ LED structure on (0001) GaN bulk crystals with a threading dislocation density of 10^6^ cm^–2^. (ii) Shallow ion implantation to define the current aperture. This was achieved by a high-dose (*D* = 3 × 10^13^ cm^–2^) implantation of He ions, effectively increasing the resistivity of the TJ (and the layers above) and preventing undesired current flow. Notably, the region where the μLEDs were formed was covered by a photoresist mask and remained unimplanted. (iii) Regrowth of 200 nm highly doped GaN/Si. (iv) Deposition of metallic contact (Ti/Al/Ni/Au) for both top and bottom contacts. (v) Mesa etching to isolate chips with single μLEDs after regrowth. More details about epitaxial growth and fabrication process can be found in the previous work.^19^

The energy band diagram for the TJ LED structure under an operating voltage of 3.2 V, presented in Figure 2b, was obtained using a 1D DDCC solver, which uses the Poisson-Schrödinger-drift-diffusion model.^34^ On the left side of the graph shows the LED region with a single QW, where radiative recombination occurs. Moving further to the right, there is the TJ region, which is polarized in the reverse direction. In this way, electrons from the valence band tunnel into the conduction band in the TJ region.^35^

The light-current–voltage characteristics of μLEDs were examined by using a source meter unit (Keithley SMU2400) and a power meter (Thorlabs S130C). Numerous μLEDs with diameters of 2, 5, and 10 μm were measured, and representative devices were selected for further analysis. The EL of each device was measured at various current densities during continuous-wave operation—very low (approximately 2.5–10 A/cm^2^, except smallest μLED, where optical power was below charge-coupled device (CCD) detection limits for *j* < 50 A/cm^2^), medium (approximately 200–500 A/cm^2^) and high (approximately 1000 A/cm^2^). To investigate the lateral carrier diffusion, we analyzed the relative carrier distribution within an active region by observing the spatial distribution of the EL with a Gaussian beam telescope. This setup includes a sample holder, an aspherical flat-convex lens (focal length = 4.5 mm), a convex-flat lens (focal length = 300 mm), filters to adjust the measured intensity range, and a CCD camera. Further details of this measurement approach can be found in the work of Rogowsky et al.^36^

The distribution of carriers inside the QW of μLEDs can be described by a continuity equation with a diffusion term in polar coordinates:
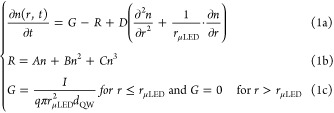
1where *D*, *G*, and *n* represent diffusion constant, generation rate, and carrier density, respectively.^37^ Recombination rate (*R*) is expressed by eq 1b, where *A*, *B*, and *C* are SRH, radiative recombination, and Auger–Meitner coefficients, respectively. The generation rate is uniform and directly proportional to the applied current (eq 1c), and it occurs only in the volume of the active region under the unimplanted region, described by radius of the unimplanted area and thickness of the QW (*d*_QW_). Generation outside of this volume equals 0. The model does not account for carrier-concentration-induced drift due to screening of the polarization field. To obtain numerical solutions, we employed a finite differences method, allowing us to consecutively solve eqs 1a–c until reaching a state of steady operation .

Lateral diffusion length was defined as
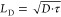
2where τ is the effective carrier lifetime.^38^ The reciprocal of the effective carrier lifetime was obtained from the calculated carrier density and *A*, *B*, and *C* coefficients as
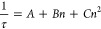
3The carrier lifetime, hence recombination rates, are current-dependent.^33,39,40^

## 3 Results and Discussion

**Figure 3:**
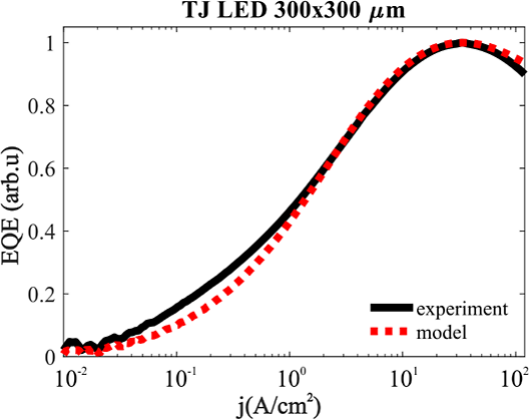
Measured (experiment) and fitted (model) dependence of EQE on current density for a standard size LED (300 × 300 μm^2^) processed from the same wafer as μLEDs.

First, we determined the *A*, *B*, and *C* coefficients based on the measurement of the standard 300 × 300 μm^2^ TJ LED, in which the influence of diffusion is negligible. Figure 3 illustrates the measured external quantum efficiency (EQE), for a 300 × 300 μm^2^ TJ LED originating from the same wafer as the μLEDs. We obtained the following fitted coefficients: *A* = 1.9 × 10^6^ s^–1^, *B* = 4 × 10^–14^ cm^3^ s^–1^, and *C* = 1 × 10^–34^ cm^6^ s^–1^. It is important to note that the choice of *A*, *B*, and *C* parameters is not unique. It is well-known that the same fit can be obtained with different sets of *A*, *B*, and *C* and a large span of parameter values were used throughout the literature. The sole reason why these particular values of recombination coefficients were used is because they lie on the lower end of the range found in the literature. We will see that the particular values have an effect on the diffusion coefficient. We will come back to this issue in the last section of this paper by discussing the influence of a different set of *A*, *B*, and *C* parameters.

**Figure 4:**
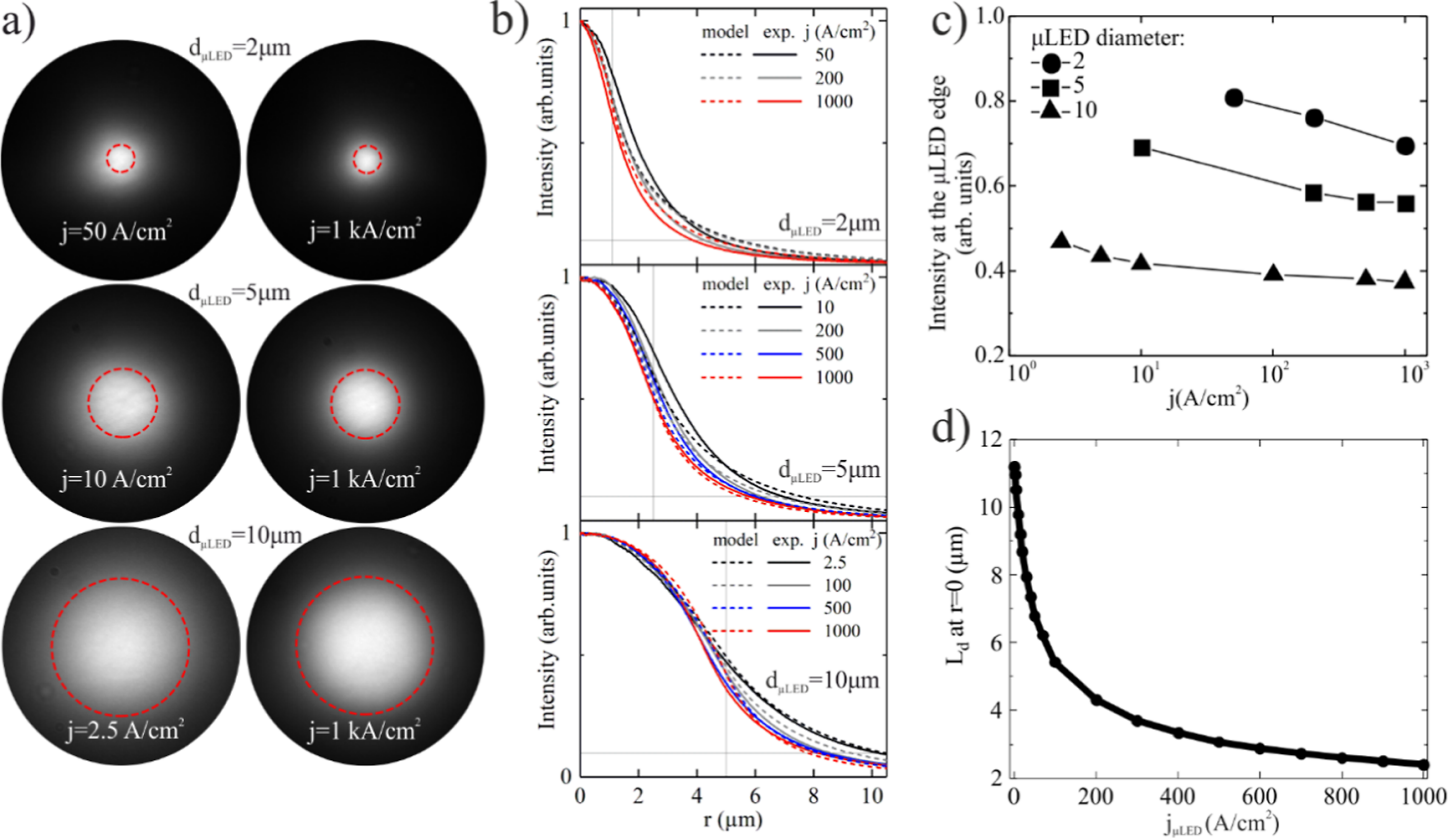
(a) CCD images of μLEDs with diameters (*d*_μLED_) of 2, 5, and 10 μm at two current densities: the smallest value at which the light is detectable, and at 1 kA/cm^2^. (b) Calculated intensity profiles for 2, 5, and 10 μm μLED as a function of the distance (*r*) from the center of the μLED, presented together with experimental data (solid lines). Vertical and horizontal gray lines represent μLED radius and 10% of maximum intensity, respectively. (c) Intensity at the edge of implanted region defining the μLED size [dashed red circle in (a)]. (d) Dependence of lateral diffusion length on current density for the device with a diameter of *d*_μLED_ = 10 μm.

Second, we investigated the emission intensity profiles of μLEDs. CCD images in Figure 4a illustrate the light intensity distribution from ion-implanted μLEDs under the lowest and highest measured current densities. In the images, the red dashed lines indicate the unimplanted area for each respective device. To provide more quantitative insight, we present in Figure 4b the measured light intensity profiles obtained by the arithmetic average of cross sections through the central point of the μLED. The intensity profiles, representing emitted light from the device, are proportional to the radiative rate, which, in turn, scales with *n*^2^. Additionally, the calculated light emission profiles, also derived as *n*^2^, are presented in Figure 4b. The experimental and calculated intensity profiles are depicted as a function of the distance (*r*) from the center of the μLED. The widest distribution is observed at lower current densities, and it gradually narrows as the current density increases, similarly to the experimental data. Qualitative analysis of these profiles reveals that (i) the light emission area expands beyond the unimplanted area, (ii) the light emitting area is changing with the current density, it is noticeably larger at lower current densities, and (iii) the intensity of the emission within the defined unimplanted area is not constant. It is highest at the center of the μLED and begins to decrease moving outward from the center. The last two findings can be well observed in Figure 4c, which shows the dependence of light intensity at the edge of the current injection area (unimplanted area).

Next, we numerically solved eq 1 to fit the calculated emission profile to the data presented in Figure 4b. Given a predetermined set of coefficients *A*, *B*, and *C*, from the 300 × 300 μm^2^ device, the only fitting parameter in eq 1 is a diffusion constant. The most optimal parameter to fit experimental data was determined to have a value of *D* = 2.5 cm^2^ s^–1^.

We report on the *L*_D_ calculated for carrier density in the center of μLED. The dependence of *L*_D_ on current density is shown in Figure 4d. *L*_D_ reaches approximately 11.2 μm for *j* = 1 A/cm^2^ and reduces to 2.4 μm for *j* = 1000 A/cm^2^.

**Figure 5:**
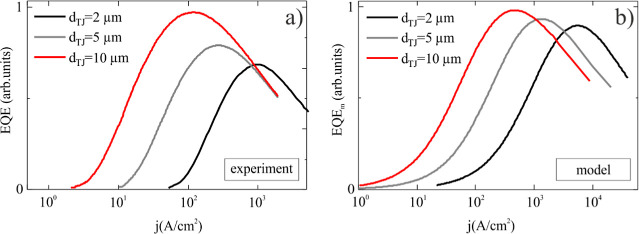
(a) EQE as a function of current density, measured in the experiment (b) and calculated using the model.

Figure 5a illustrates the dependence of EQE on current density, which is defined by the ratio of the optical power (*L*) and current (*I*). It reveals a drop of maximum EQE for smaller μLED, accompanied by a shift of *j*, at which the maximum of EQE occurs, toward higher values as the diameter of the μLED decreases. A similar effect is present also in standard μLED and is attributed to the increase of nonradiative recombination on the sidewalls of μLEDs. However, the size of the ion implanted μLEDs is defined by the electrical path limited by the size of the unimplanted TJ. The active QW region remains undisturbed by factors that typically lead to an increase in nonradiative recombination in standard μLEDs. As a result, the typical explanation cannot be used in this situation. In the next paragraph, we will see that the diffusion model can be used to explain the dependence of EQE on size of the devices.

In Figure 5b, we show the modeled dependence of the EQE (denoted as EQE_m_) on current density. EQE_m_ curves are obtained by trapezoidal numerical integration of *n*^2^ assuming a circular symmetry. As can be seen, the EQE_m_ curves present size-dependent behavior, similar to the experimental ones. Two effects can be noticed. First, the maximum of EQE_m_ drops with decrease of the μLED size and second, current density, at which the maximum of EQE_m_ is observed, increases. It turns out that these two effects are codependent and result from the lateral diffusion of carriers. We will now discuss the origin of such a behavior in detail. As shown in Figure 4b, the emission intensity and therefore the carrier density are highest in the center of the circular μLED and decrease further away from the center due to the lateral diffusion of carriers. Importantly, the distance to which the carriers diffuse with respect to the edge of the injection area is comparable between devices of different size. Therefore, the smaller the size of the μLED injection area, the higher the part of carriers recombining outside of the injection area. The size dependence in EQE comes from the fact that, for a given current density, in the case of a smaller μLED injection area the average carrier density (inside and outside of the injection area) will be lower. Low carrier density leads to carrier recombination through the nonradiative SRH process. This causes the shift of the maximum of EQE to higher current densities. Furthermore, the fact that fewer carriers recombine radiatively results in a reduction of the maximum value of EQE. Thus, the change in the spatial distribution of carrier density within the μLED area, caused by lateral carrier diffusion, results in size-dependent variation in efficiency of implanted μLEDs. It is important to notice that, while the trend of both the decrease of maximum EQE value and shift of current density at which the maximum for smaller μLED size is observed in both the model and the experiment, it needs to be acknowledged that the model does not reproduce the experimental results in quantitative terms. This shows that more detailed modeling is necessary to fully reproduce the experimental data. We discuss the effect of drift of electrons and holes in opposite directions as one of the missing elements of the model in Supporting Information.

**Figure 6:**
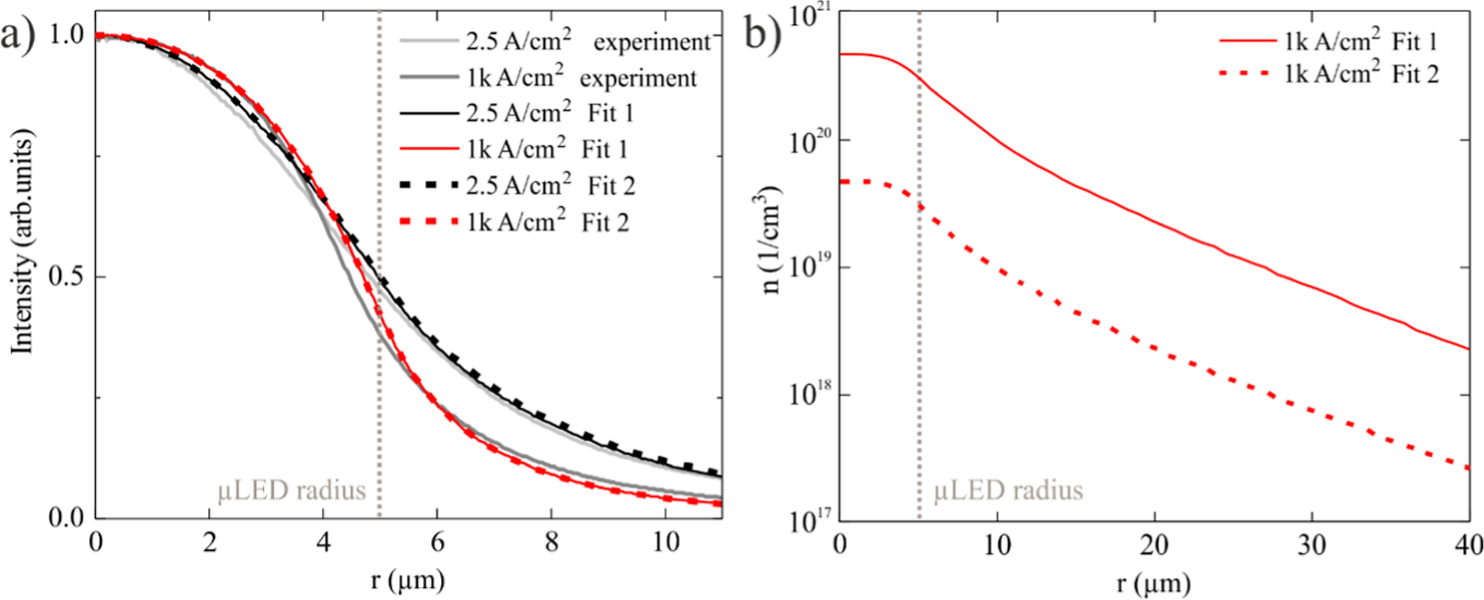
(a) Intensity profiles for 10 μm μLED. Solid, dotted, and gray lines represent Fit 1, Fit 2, and experimental data, respectively. (b) Calculated carrier density distribution of 10 μm μLED for Fits 1 and 2.

As said earlier, the choice of values of *A*, *B*, and *C* parameters to fit the EQE for large LED in Figure 3 is not unique. Initially, we used *A*, *B*, and *C* coefficients with very low values, referred as Fit 1 in Table 1. An intriguing observation arises when the experimental data are fitted using an alternative set of ABC parameters, which are more prevalent in the existing literature (see Fit 2 in Table 1). These coefficients yield the same dependence of EQE on current density, as presented in Figure 3. In Figure 6a, we present fitted intensity profiles for 10 μm μLEDs at both low (2.5 A/cm^2^) and high (1000 A/cm^2^) current density for Fits 1 and 2. Both the intensity profiles and EQE vs current density can be well reproduced with both sets of coefficients. Notably, Fit 2 results in a much higher diffusion constant of 25 cm^2^ s^–1^, which exceeds values typically reported in the literature.^29,41^ However, Fits 1 and 2 differ in their predicted carrier density. Specifically, at *j* = 1000 A/cm^2^, Fit 1 yields a carrier density of 7 × 10^20^ cm^–3^, while Fit 2 predicts a carrier density of 8 × 10^19^ cm^–3^ (Figure 6b). In this study, to characterize the experimental data, a choice must be made between embracing a notably high diffusion coefficient or, alternatively, decreasing the values of coefficients *A*–*C*, thereby inducing a higher carrier density.

**Table 1:** Fitting Parameters for 300 × 300 μm LED

	*A* (s^–1^)	*B* (cm^3^ s^–1^)	*C* (cm^6^ s^–1^)	*D* (cm^2^ s^–1^)	*n* (cm^–3^) at *j* = 1 kA/cm^2^
Fit 1	1.9 × 10^6^	4 × 10^–14^	1 × 10^–34^	2.5	7 × 10^20^
Fit 2	1.9 × 10^7^	4 × 10^–12^	1 × 10^–31^	25	8 × 10^19^

This initiates a discussion of recombination and diffusion constants for high-quality InGaN active layers. If the ABC parameters align with majority of the prior literature, then *D* can possibly reach tens of cm^2^ s^–1^. Conversely, lower *ABC* and *D* coefficients (Fit 1) lead to a higher carrier density. This highlights the necessity for further research into InGaN material properties to enhance the μLED efficiency and performance.

## 4 Conclusions

To summarize, we have fabricated and studied ion-implanted ultrasmall μLEDs with diameters 2, 5, and 10 μm, highlighting the influence of lateral carrier diffusion on their performance. The emission intensity profile was explored using EL measurements by a Gaussian beam telescope. We observed a significant expansion of the light emitting area over the defined μLED size. Furthermore, we observe a μLED size dependent change in the EQE. In order to understand this behavior, we applied a diffusion model of carriers within the QW. This investigation revealed that both size-dependent efficiency and enlargement of the emission area can be attributed to lateral carrier diffusion inside the QW. Furthermore, the diffusion length was found to be dependent on current density, with lower densities leading to larger light-emitting areas. At the smallest current density in the order of 1 A/cm^2^ diffusion length was determined to be 11.2 μm and decreased down to 2.4 μm for *j* = 1000 A/cm^2^.

Moreover, we discussed the intriguing consequences of the values of fitting parameters used in the modeling. Two different sets of *ABC* parameters, both of which can reproduce the experimentally measured EQE of the standard size LED (300 × 300 μm^2^), were investigated. The diffusion constant, which is used to model the μLEDs, depends on the values of the *ABC* parameters. We show that, depending on the used *ABC* parameters, the diffusion constant can range from 2.5 up to 25 cm^2^ s^–1^. In the case of the lower limit of *D* = 2.5 cm^2^ s^–1^, which is commonly accepted in the literature, the *ABC* parameters are surprisingly low, which leads to an unrealistic carrier density of up to 7 × 10^20^ cm^–3^. On the other hand, the upper limit of *D* = 25 cm^2^ s^–1^ is obtained for commonly used *ABC* parameters, which yield commonly accepted carrier densities. However, such a high value of the diffusion coefficient was never reported. Whether the diffusion constant in the plane of InGaN QWs is high or the ABC parameters are low remains an open question. These findings emphasize the need for further exploration of material properties for high-quality InGaN layers for optimizing the efficiency and performance of μLEDs.

## Supporting Information Available

Brief discussion on the influence of drift on the lateral distribution of carriers and the influence of absorption in implanted regions on emission profiles (PDF)
