# Supplementary material for: Lateral Carrier Diffusion in Ion-Implanted Ultra-Small Blue III-Nitride MicroLEDs
Source: ACS Appl Mater Interfaces. 2025 Jan 15;17(4):6473–9. doi: 10.1021/acsami.4c14784 (PMC11788977; doi:10.1021/acsami.4c14784)
Supplement: Supplementary file 1 — am4c14784_si_001.pdf [file am4c14784_si_001.pdf]

Supplementary information:

1) The influence of drift on the lateral distribution of carriers

In InGaN/GaN active regions there are two primary causes of the lateral carrier transport. The first is diffusion, which arises from differences in carrier concentrations. The second is drift, which results from variation in potential energy within the energy landscape.

Quantum Confined Start Effect (QCSE), originating from internal polarization fields at the interfaces of nitride alloys, introduces high internal electric field leading to the spatial separation of carrier wavefunctions. It was shown that QCSE leads to higher diffusion length, as electron and hole overlap is smaller, hence carriers are more likely to diffuse than recombine.[1] In terms of lateral carrier transport, when carriers are injected into the device, piezoelectric fields become shielded. This results in a shift in the potential landscape within the carrier injection volume, thereby creating lateral electric fields.[2] However, when there is applied voltage, the effect of different potential in the area inside and outside of vertical current flow (inside and outside µLED in Fig. S1a) dominates the direction of electric field.

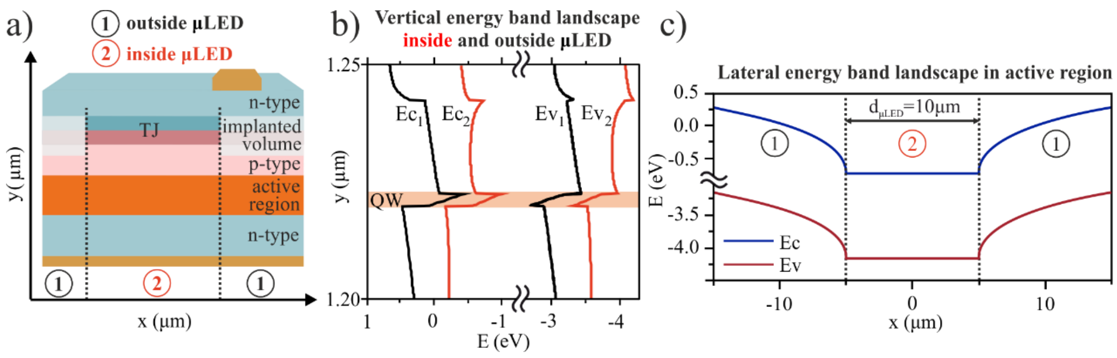

Figure S1. (a) Schematic representation of the µLED with marked regions located both inside and outside and µLED. (b) Energy band landscape parallel to $\vec{c}$ axis (c) and at y=1.221 µm (position of the centre of the QW) and perpendicular to $\vec{c}$ axis for a 10µm µLED.

The drift term, represented as $D\frac{q}{kT}\nabla nE$, where E corresponds to lateral electric field, should be added to continuity equation as follows:

$$\begin{cases} \dfrac{\partial n(r,t)}{\partial t} = G - R + D\left(\dfrac{\partial^2 n}{\partial r^2} + \dfrac{1}{r_{\mu LED}} \cdot \dfrac{\partial n}{\partial r}\right) + D\dfrac{q}{kT}\nabla nE, & \text{(1a)} \\ R = An + Bn^2 + Cn^3 & \text{(1b)} \\ G = \dfrac{I}{q\pi r_{\mu LED}^2 d_{QW}} \quad for\ r \le r_{\mu LED}\ and\ G = 0 \quad for\ r > r_{\mu LED} & \text{(1c)} \end{cases}$$

However, we could not obtain a fit better then without the drift term. To provide further insights, we chose to simulate carrier behaviour in µLEDs using a 2D DDCC solver.[3] Since the tunnelling effect is beyond the solver's capabilities, we conducted simulations using a standard µLED structure, wherein the current aperture was defined by the size of the p-type contact, corresponding to the size of the un-implanted region.

The carriers are generated only within the volume defined by the un-implanted region. Consequently, a significant carrier density gradient is present, which influences the energy band landscape and set the direction of lateral electric field for both electrons and holes. With higher current densities, a stronger screening effect occurs, leading to a change in the bandgap and, subsequently, the generation of a larger electric field, as depicted in Fig. S1b. The lateral energy landscape of 10µm µLED is illustrated in Fig. S1c. Notably, the conduction band energy within µLED volume is lower than the energy of the conduction band outside this volume. As a consequence, electrons tend to accumulate preferentially inside of the µLED volume. Conversely, for holes, the energy of the valence band is higher in the µLED volume, prompting their movement outside of this volume. Due to the distinct behaviours exhibited by holes and electrons, a definitive direction for the lateral electric field within our model, assuming equal densities of holes and electrons, cannot be unambiguously established. The study would call for further improvements to the transport model.

2) The influence of absorption in implanted regions on emission profiles

In order to quantify absorption impact on our devices we measured light transmittance at λ = 456 nm for both the implanted and unimplanted regions of the µLED. For reference, we also measured the transmittance in area in between devices, which does not contain the epitaxial structure i.e. only substrate. The transmittance was 63.3%, 63.9% and 66.7% for the implanted, unimplanted and substrate regions, respectively.

As can be seen the light absorption due to implantation damage is 1% (1-63.3/63.9), which is too small to be noticeable in the intensity profile. Such a small absorption is due to a small thickness of the implanted layer. Therefore, the influence of absorption on emission profiles is negligible.

Table S1*. Averaged optical power values measured after the sample. Laser source: 115 mA, 5.65 V, 20 C, optical power: 8.25 mW.*

	Optical power (mW)
Unimplanted region	5.22
Implanted region	5.27
Substrate region	5.44

(1) Shen, H.-T.; Weisbuch, C.; Speck, J. S.; Wu, Y.-R. Three-Dimensional Modeling of Minority-Carrier Lateral Diffusion Length Including Random Alloy Fluctuations in (In, Ga) N and (Al, Ga) N Single Quantum Wells. *Physical Review Applied* **2021**, *16* (2), 024054. DOI: 10.1103/PhysRevApplied.16.024054.
(2) Danhof, J.; Solowan, H. M.; Schwarz, U. T.; Kaneta, A.; Kawakami, Y.; Schiavon, D.; Meyer, T.; Peter, M. Lateral charge carrier diffusion in InGaN quantum wells. *physica status solidi (b)* **2012**, *249* (3), 480-484. DOI: 10.1002/pssb.201100476.
(3) Wu, Y.-R. *DDCC 2D*. 2023. http://yrwu-wk.ee.ntu.edu.tw/index.php/ddcc-2d/ (accessed 10/2023)
